# Nutritional status among adolescents in India: A school-based retrospective cohort study

**DOI:** 10.6026/973206300222262

**Published:** 2026-04-30

**Authors:** Arti Gupta, Praveen Kumar S, Srineetha Paruchuri, Venkatashiva Reddy B, Navya Krishna Naidu, Rajeev Aravindakshan, Mounika Kollimarla

**Affiliations:** 1 Department of Community & Family Medicine, All India Institute of Medical Sciences, Mangalagiri, Andhra Pradesh, India

**Keywords:** Adolescent, anaemia, body mass index (BMI)

## Abstract

Adolescent anaemia and malnutrition remain a major public health challenges in India Adolescence, yet district-level longitudinal evidence is limited. (10-19 years) is a nutritionally demanding period with high risk of anaemia and malnutrition. Therefore, it is of interest to assess baseline prevalence and follow-up changes in haemoglobin and nutritional status among adolescents enrolled in government and private schools of Guntur district during 2023-2025. At baseline, 6,175 adolescents were assessed, with an anaemia prevalence of 64% and a dual burden of malnutrition was observed, with 14% thin/severely thin and 24% overweight/obese. Among 618 adolescents successfully followed up, McNemar's test demonstrated statistically significant worsening transitions for both anaemia (odds ratio = 3.3) and BMI status (odds ratio = 0.27). Strengthening longitudinal school health registries and sustained nutrition-specific interventions are recommended.

## Background:

Anaemia remains one of the most prevalent nutritional deficiencies worldwide and contributes substantially to impaired growth and development [1]. WHO also highlights menstruating adolescent girls among the groups most vulnerable to anaemia, reflecting the combined effects of biological demand and nutrition-infection interactions [2]. Global anaemia prevalence among key groups, 30.7% of women aged 15-49 years and 39.8% of children aged 6-59 months were affected, highlighting a persistent global burden [3]. The pooled prevalence of anaemia among Indian adolescent girls has been reported to be approximately 65%, with regional variation [4]. According to the National Family Health Survey (NFHS-5), 59.1% of adolescent girls (15-19 years) and 30.6% of adolescent boys are anaemic; these patterns highlight the need for strengthened, context-specific strategies to address adolescent anaemia in India [5]. In Andhra Pradesh, NFHS-5 data reveal anaemia prevalence of 32.4% among adolescent boys and 50.47% among girls, reflecting persistent nutritional vulnerabilities amid statewide interventions, such as the Anaemia Mukt Bharat strategy, which involves weekly iron-folic acid supplementation [6]. The rationale for the present study arises from two linked considerations: adolescence is a time of high iron demand and of habit formation in dietary practices; untreated anaemia undermines educational attainment, productivity and future maternal-child health [7]. Alongside anaemia, adolescents in South Asia and many low- and middle-income countries are facing a substantial burden of low body-mass index (thinness/underweight), which coexists alongside rising overweight in some populations [8]. Recent pooled analyses and reviews indicate persistent levels of adolescent under nutrition across the region, with marked subnational heterogeneity and particularly high prevalence among girls and marginalized communities [9, 10]. Low BMI during adolescence is associated with impaired linear growth, reduced muscle mass and physical capacity, delayed pubertal development and poorer educational and economic outcomes in later life and it may exacerbate susceptibility to micronutrient deficiencies such as iron deficiency [11]. While the NFHS defines the broad scale of the problem, there is limited, contemporary, peer-reviewed evidence describing the district-level prevalence of anaemia and its nutritional correlates among adolescents in many parts of India, including districts of Andhra Pradesh [12]. Therefore, it is of interest to describe the prevalence and longitudinal changes in anaemia and nutritional status among adolescents in Guntur district.

## Materials and Methods:

The study aimed to estimate the prevalence of anaemia and examine its association with nutritional indicators and selected socio- demographic factors. The objectives of the study are to estimate the baseline prevalence of anaemia and malnutrition among adolescents enrolled in government and private schools in Guntur district Andhra Pradesh, India and to determine changes in haemoglobin levels and malnutrition between baseline and follow-up among adolescents successfully followed up during 2023-2025. A retrospective school-based cohort study was conducted after getting Institutional Ethics Committee approval (AIIMS/MG/IEC/2024-25/471). No personal identifiers were received and a waiver of consent was obtained. Routine school health camps and outreach visits were conducted between 2023 and 2025 across government and private schools in Guntur district (Mangalagiri, Duggirala, Kolakaluru, Tenali and Chilakaluripet Mandal). Health services consisted of clinical examination, anthropometric measurements using a calibrated seca 213 weighing scale and a SECA 813 stadiometer and hemoglobin measurement using a mission plus Hb (ACON) point-of-care hemoglobinometer. Health services were provided to students by a team from the centre for rural health AIIMS (CRHA) (a peripheral unit of All India Institute of Medical Sciences {AIIMS}, Mangalagiri), PHC Nutakki, which consists of junior resident, Interns, MNO/FNO (Male Nursing Orderly/ Female Nursing Orderly) with supervision from senior residents and faculty. The students' health details were maintained in a separate adolescent register. The data from registers were entered by interns in separate Google sheets for each school; the validity of the entered data was frequently verified by residents and faculty. Data retrieved from Google sheets was compiled into a single master sheet. The sheets had health data of baseline visit and visit 2, during 2023-2025 of 37 schools/ junior colleges, at baseline, 6175 observations were retrieved and 618 follow-up observations were retrieved. The study population comprised students aged 10-19 years enrolled in the participating schools. All students who completed anthropometric measurements and haemoglobin assessment were included, while those with common chronic conditions known to affect growth, viz., hypothyroidism and moderate-to-severe congenital heart disease, were excluded. The master Google sheet was taken for analysis in R 4.5. Anthropometric z-scores (BMI-for-age) were calculated using the WHO 2007 growth reference for 5-19 years using WHO AnthroPlus/CRAN. BMI-for-age z-scores were classified using WHO cut-points (severe thinness, thinness, normal, overweight, obesity) [13]. Haemoglobin (g/dL) was analysed both as a continuous variable and as a categorical outcome using WHO haemoglobin cut-offs by age and sex to define anaemia [14]. Continuous variables were summarised as mean and standard deviation, or median and interquartile range, based on normality and categorical variables were summarised as frequencies and proportions. The hemoglobin changes between the 1st and 2nd visits were tested using McNemar's test and the within-subject, categorical changes in BMI between the 1st and 2nd visits were determined using Bowker's test; statistical significance was defined as p < 0.05.

## Results and Discussion:

A total of 6175 students were enrolled in Baseline. Most of the students were in the 10-13-year age group, accounting for three-fifths of the cohort at the beginning and female students (63%) were more than male students (47%). Among the enrolled students, most were from government schools (65%) and the remaining from private schools and colleges (35%). The prevalence of anaemia at the baseline visit was 64%. Anthropometric assessment at baseline showed a mean height of 150cm and a mean weight of 44kg. BMI assessment at baseline revealed a dual burden of malnutrition. The majority (62%) of adolescents had a normal BMI-for-age, 14% were classified as thin/severely thin and 24% as overweight/obese (Table 1 - see PDF). Among 2,468 adolescents, anaemia was significantly associated with age, gender, institute type and BMI (p < 0.05). Age-category-wise analysis showed a progressive increase in anaemia prevalence with age, rising from early adolescence [10-13 years] (46%) to late adolescence [17-19 years] (64%), indicating a significant (p-value < 0.001) cumulative nutritional deficit with age. Gender-based differences were significant (p-value < 0.001), with a higher prevalence of anaemia in adolescent girls (56%) than in boys (44%). Anaemia status was also significantly associated with the type of institution (p-value < 0.001). Adolescents attending private schools had a higher prevalence of anaemia (69%) than those attending government schools (38%). Anaemia and BMI-for-age showed a statistically significant association (p-value 0.007). Prevalence of anaemia was higher among adolescents with normal BMI (55%) and the prevalence was similarly high in the overweight/obese category (57%). In contrast, a lower prevalence of anaemia (46%) was observed in the thinness or severe thinness category. In the McNemar's test to determine binary (Anemic versus not anemic) change between baseline and visit 2 was significant (p value), with an odds ratio of 3.3, indicating transitions are worsening rather than improving. Discordant pairs, anaemic to non-anaemic were 172 and non-anaemic to anaemic were 171. In the transition matrix of the BMI category between baseline and visit 2, movement occurred between categories. Where 62% normal BMI in Baseline shifted to 32% in visit 2, 14% underweight/thinner BMI in baseline shifted to 59% in visit 2 and 24% overweight/obese BMI in Baseline shifted to 9.6% in visit 2. In the McNemar's test to determine binary (normal versus not normal) change between baseline and visit 2 was significant (p value), with an odds ratio of 0.27, indicating transitions are worsening rather than improving. Discordant pairs, anaemic to non-anaemic, were 47 and non-anaemic to anaemic were 171 (Figure 1 - see PDF). The present study documents adolescent nutrition and anaemia among school-going children.

A similar cross-sectional study conducted in the urban area of Guntur reported a high prevalence of undernutrition (43%) and overnutrition (17%), highlighting the coexistence of both forms of malnutrition among adolescents [15]. While such cross-sectional evidence provides a snapshot of the nutritional burden, the present study adds longitudinal insights. At baseline, a significant number of adolescents were undernourished and nearly two-thirds were anaemic. At follow-up, underweight prevalence increased, anaemia decreased, indicating overall limited improvement. These findings show that adolescent nutrition and anaemia are not static issues. They change over time, influenced by factors such as rapid growth during puberty, dietary habits, infections and the quality of school health programs [16]. The rise in underweight status suggests that current interventions may have lacunae in reaching or effectively supporting all adolescents. Key challenge was the practical difficulties in maintaining consistent contact with adolescents over time, limiting the effectiveness of health programs and research. Indian cohort studies like UDAYA suggest that factors such as menstruation, diet and access to healthcare play a role in these differences [17]. Indian data demonstrate that a substantial fraction of adolescent anaemia is attributable to non-iron-only causes, including folate/vitamin B12 deficiency and anaemia of other causes, with dimorphic patterns also common. This matters because haemoglobin may not sustainably improve if interventions focus narrowly on iron while diets remain poor in animal-source foods, pulses and overall diversity [18]. Awareness campaigns targeting families and communities on adolescent nutrition and the risks of anaemia can drive demand for healthier diets and greater access to healthcare. Policies that incentivize local production and availability of nutrient-rich foods can improve sustainable dietary improvements. A mild reduction in anaemia at visit 2 may plausibly reflect short- term improvements in programme exposure (e.g., health talks, health-camp screening/referral, or seasonal variation in diet and infections). Qualitative work from India highlights gaps in awareness, tablet supply and engagement that can undermine sustained impact [19]. Digital tools and mobile health technologies should be explored to improve follow-up and data collection, reducing losses in longitudinal tracking. Expanding social support programs to include adolescent nutrition can address underlying socioeconomic barriers. Systematic reviews indicate that weekly IFA-based strategies can improve iron status/haemoglobin outcomes and emerging evidence links intermittent IFA exposure to better cognitive outcomes in adolescent settings, which strengthens the school-based rationale [20]. Investment in training teachers and health workers to recognise and respond to under nutrition and anaemia is critical. Empowering schools to act as nutrition hubs can increase the reach and effectiveness of interventions.

## Conclusion:

We show the need to integrate adolescent nutrition more firmly into broader health and education agendas. School health programs should not operate in isolation but should link closely with community and primary healthcare systems to comprehensively address nutrition and anaemia. Policies must encourage multi-sectorial collaboration among education, health, agriculture and social protection to improve dietary diversity and food security for adolescents. A focus on adolescent nutrition today is an investment in the health and productivity of future generations.

## Advancement to knowledge:

This study provides district-level longitudinal evidence on adolescent anaemia and BMI transition in Andhra Pradesh. The study captures the individual nutritional transitions over time. The findings demonstrate worsening nutritional trajectories despite existing national programmes, highlighting the need for sustained, longitudinal school-based monitoring.

## Ethics consideration and funding:

The study was conducted after getting Institutional Ethics Committee approval (AIIMS/MG/IEC/2024-25/471), dated 28 November 2025. No personal identifiers were received and a waiver of consent was obtained.
